# Diversity and conservation of the genome architecture of phages infecting the Alphaproteobacteria

**DOI:** 10.1128/spectrum.02827-23

**Published:** 2023-11-22

**Authors:** Jonathan R. Hyde, Thomas Armond, Jacob A. Herring, Sandra Hope, Julianne H. Grose, Donald P. Breakwell, Brett E. Pickett

**Affiliations:** 1 Department of Microbiology and Molecular Biology, Brigham Young University, Provo, Utah, USA; Thomas Jefferson University, Philadelphia, Pennsylvania, USA

**Keywords:** bacteriophages, Alphaproteobacteria, comparative genomics, phylogenetic reconstruction, protein orthologs, clusters

## Abstract

Bacteriophages are viruses that are only capable of replicating inside a suitable bacterial host cell. Here, we performed a comprehensive meta-analysis of all 103 publicly available phage genomes that are known to infect members of the Alphaproteobacteria. We combined the sequence data and associated metadata with results from various comparative genomic and phylogenetic methods to quantify gene presence, families of orthologous gene products, and assign the phages into clusters and subclusters. Comparative genomic analyses, such as this one, allow differentiation of phages based on their evolutionary relatedness. The results of our analyses justify the classification of these phages into 16 clusters and 6 subclusters to ideally balance sequence diversity, protein orthologs, and core genes with host range. This study is the first to group phages infecting the Alphaproteobacteria. We expect that these results will inform future studies and that our classification schema will improve the classification of phages that infect bacterial taxa in the Alphaproteobacteria class.

**IMPORTANCE:**

This study reports the results of the largest analysis of genome sequences from phages that infect the Alphaproteobacteria class of bacterial hosts. We analyzed over 100 whole genome sequences of phages to construct dotplots, categorize them into genetically distinct clusters, generate a bootstrapped phylogenetic tree, compute protein orthologs, and predict packaging strategies. We determined that the phage sequences primarily cluster by the bacterial host family, phage morphotype, and genome size. We expect that the findings reported in this seminal study will facilitate future analyses that will improve our knowledge of the phages that infect these hosts.

## INTRODUCTION

Bacteriophages are obligate intracellular parasites that rely on the resources of a host bacterial cell in order to replicate. The Alphaproteobacteria are either intracellular, facultative, or free-living prokaryotic symbionts (1). The Alphaproteobacteria class includes some of the most metabolically diverse and abundant organisms on Earth (2). Phylogenetic analysis has divided this class into three bacterial subclasses (3). First, the Caulobacteridae include the phototrophic Rhodospirillales, nitrogen-fixing Rhodobacterales (4, 5), the symbiotic and nitrogen-fixing Rhizobiales, and the Caulobacterales. Second, the Rickettsidae subclass includes the obligate intracellular parasites *Rickettsia* spp., *Wolbachia* spp., protomitochondrion (3), and the Pelagibacteriales (6). Finally, the nitrogen-fixing Magnetococcidae, which produce magnetosomes for orientation, are included (7).

Examining the genomes of phages that infect Alphaproteobacteria provides insights into how these entities are related to each other and to their hosts, predator-prey relationships (8), and the potential for phage therapy (9). More fundamentally, such analyses can improve our understanding of their evolution (10, 11), molecular biology (10 – 13), mechanisms of genetic recombination and horizontal gene transfer (1, 14 – 16), and the spread of virulence factors (17). It also sets a framework for the comparison of other phage genomes as they are sequenced and characterized. Even though the study of phages has greatly contributed to our understanding of biology, phage diversity is remarkably underestimated due to a paucity of whole genome sequencing data and the associated annotations.

Recent past studies of phage genomes focused on those that infect mycobacteria (14, 18), Enterobacteriaceae (19, 20), *Bacillus* (21, 22), *Propionibacterium acnes* (23), and *Paenibacillus larvae* (24). Incorporating complementary visualization tools and metrics such as sequence dot plots, average nucleotide identity (ANI), and annotation tools such as Phamerator has been shown to improve the robustness of the results (25). These data can be used to predict protein orthologs to enable the grouping of phages into superclusters, clusters, and subclusters. A supercluster is composed of phages whose genes, while lacking nucleotide sequence identity, encode proteins with similar functions (19). Superclusters are usually named after the phage that was first identified or the genome of those that were first analyzed (19). Similarly, genomes sharing >50% cumulative sequence identity and gene synteny are defined as a cluster (19), whereas more closely related genomes form subclusters.

Pope et al. used various quantitative indices to better characterize the diversity of phages that infect mycobacteria (18). Genes with common functions were used to compare phage genomes within and between clusters and subclusters. These indices included “Cluster Averaged Shared Phamilies” (CLASP), the Cluster Associated Phamilies (CAP) index, and the Cluster Cohesion Index (CCI). The major capsid protein is commonly used to initially identify related phages, while phylogenies of the large terminase gene product can predict putative capsid packaging strategies (19, 26, 27). Diversity within phage clusters indicates not only an ancient evolutionary past but also that these genomes are found in dynamic populations. For example, a study grouped ~600 genomes of phages that infect mycobacteria into more than 30 clusters and 50 subclusters (18) and found genes and modules of genes in both related and unrelated phage clusters.

The current study characterizes 103 genomes of phages that infect Alphaproteobacteria from GenBank (28, 29). Using a variety of analytical methods, we propose 3 superclusters, 16 clusters, and 6 subclusters that define phages infecting the Alphaproteobacteria. To our knowledge, this is the largest analysis of phages that infect this taxon of bacteria.

## MATERIALS AND METHODS

### Compilation of the sequence database

The phage genomes compared in this study were retrieved from GenBank based on the existing literature. To ensure that most, if not all, of the relevant phages were included, the terminase gene from each phage was used as input to the preset function of TBLASTN (30) to recover any additional phages with genomes that were not found in the initial search. Manual manipulation of the initial gene in these circular genomes was performed to facilitate dot-plot analysis of the genomes and comparison of gene synteny. Coding regions in phage genomes were annotated with DNAMaster (https://phagesdb.org/DNAMaster/) (31) and, as necessary for downstream analyses, were rearranged by reverse complementation using Sequence Massager (http://biomodel.uah.es/en/lab/cybertory/analysis/massager.htm).

### Genome analysis

The nucleotide and amino acid dot plots were generated using Gepard (32), with a word length of either 10 or 11. Longer word lengths help reduce the background noise when comparing a large number of phages. ANI was calculated using the default settings in Kalign (33, 34). ANI is calculated by multiplying the percent identity and the length of the alignment, then dividing this product by the length of each coding region being compared; this value is then summed across all comparisons. This ANI metric calculates the percentage of nucleotides that are identical in pairwise sequence alignments of coding regions and can facilitate the delineation of bacterial species. Average amino acid identity was calculated with CoreGenes 3.0 (35) using a default BLASTP setting of 75. Briefly, CoreGenes determines the average number of proteins conserved between two phages. If cluster assignments were unclear using nucleotide dot plots, a CoreGenes value of 40% or greater between phages in a cluster confirmed the cluster assignments.

A custom Phamerator database was used to identify genes sharing a common protein function, determine GC content, identify the number of open reading frames (ORFs), and generate genome maps of each phage (24, 25). Specifically, Phamerator phage families (phams) were identified as genes that have a minimum of 32.5% sequence identity and an *E*-value of <1 × 10^−50^. Accessing these data in Phamerator enabled the calculation of the indices referred to by Pope (18). These included CLASP—the average number of shared phams in a cluster; the CAP index—a quotient of the total pham count for a cluster and the average number of genes per genome in that cluster; and the CCI, which is a percentage of the average number of genes per genome and the total number of phams in a cluster. The Proteinortho algorithm was used to complement the Phamerator predictions by calculating protein ortholog groups after translating the annotated coding region sequences for each phage (36). This analysis implemented a minimum of 32.5% sequence identity and an *E*-value of <1 × 10^−50^.

### Phylogenetic analysis of the terminase large subunit gene products

MUSCLE (37) was used to construct a multiple sequence alignment for all genomes with an annotated large subunit terminase protein sequence. This alignment was then used to generate a maximum likelihood phylogenetic tree using the LG + G8+F model in RaxML-ng (38). The tree was bootstrapped with 100 replicates, and any bootstrap value <50% was not displayed in the final tree output. Cluster annotations were overlaid on the tree using the Interactive Tree of Life tool (39).

### Motifs

The MEME (40) and FIMO (41) programs were used to locate reoccurring motifs in the phage genomes. A representative phage from each proposed cluster was scanned using MEME to identify sequences between 20 and 40 bp that were repeated throughout the genome. Sequences with an *E*-value of <10^−3^ were categorized as significant. Selected motifs were then compared inside and across the clusters using FIMO. Motifs found by FIMO with a *P*-value of <10^−3^ and a *q*-value of <0.05 were considered significant. The location of the motifs within the genome and where they sat in relation to putative ORFs were identified using DNAMaster and Phamerator.

### Packaging mechanisms

An existing method was used to determine shared phylogenetic ancestry between terminase amino acid sequences having characterized packaging strategies and the terminase sequences from the phages that infect Alphaproteobacteria (42, 43). An amino acid sequence alignment was generated with MAFFT (44), using default parameters, prior to reconstructing a neighbor-joining phylogenetic tree with MEGA (45), using default parameters and 1,000 bootstrap replicates. The tree was then manually reviewed to determine the ancestry of the terminase proteins from phages that infect Alphaproteobacteria with those that were previously characterized.

## RESULTS

We began by identifying 103 publicly available genome sequences from phages capable of infecting Alphaproteobacteria that met our query criteria (Table S1). We then averaged various characteristics of these phage genomes, including host, genome size, number of ORFs, GC content, number of tRNAs, and morphotype (Table 1).

**TABLE 1:** Summary of the basic characteristics of each cluster^*a*^

Phage cluster	Number of phage sequences	Genome size	GC%	ORFs	Shared protein orthologs (%)	Type^*a*^	Host
**A**	3	36,043.7 ± 51.4	65.4 ± 0.1	45.0 ± 1.0	42/46 (91)	N/A	Rhodobacteraceae
**B**	3	39,199.0 ± 768.2	40.0 ± 1.1	44.0 ± 3.6	32/52 (62)	P	*Candidatus Liberibacter*
**C**	26	40,587.1 ± 1161.5	48.2 ± 0.0	57.6 ± 0.8	54/59 (92)	P	*Brucella* spp.
**D**	2	39,397.5 ± 719.1	46.2 ± 0.1	45.0 ± 15.6	21/62 (34)	P	Rhodobacteraceae
**E1**	2	39,902.0 ± 465.3	62.2 ± 0.1	56.0 ± 0.0	11/57 (19)	S	Rhodobacteraceae
**E2**	1	39,283	64.9	58		S	Rhodobacteraceae
**F**	2	41,002.5 ± 1,529.5	33.0 ± 1.4	52.0 ± 9.9	18/84 (21)	P	*Pelagibacter* spp.
**G**	3	41,317.3 ± 670.9	58.8 ± 0.3	72.0 ± 1.0	52/93 (56)	P	*Sulfitobacter* spp.
**H1**	2	44,345.0 ± 213.5	55.0 ± 0.1	61.0 ± 0.0	19/99 (19)	S	Rhodobacteraceae
**H2**	1	42,093	56.4	52		S	Rhodobacteraceae
**I**	2	43,277.5 ± 2,399.2	61.1 ± 0.2	51.0 ± 5.7	20/81 (25)	P	*Caulobacter* spp.
**J**	4	45,494.8 ± 1,277.2	49.0 ± 0.0	59.8 ± 1.0	54/64 (84)	P	*Rhizobium* spp.
**K**	3	52,388.3 ± 1,735.4	56.0 ± 0.0	79.3 ± 3.8	75/82 (92)	M	*Rhizobium* spp.
**L**	2	63,090.5 ± 597.5	57.6 ± 0.4	81.5 ± 7.8	73/88 (83)	S	*Roseobacter*
**M1**	6	74,509.5 ± 602.2	48.6 ± 0.9	85.7 ± 5.2	35/178 (20)	P	*Roseobacter denitrificans*
**M2**	1	74,485	43	75		P	*Roseobacter denitrificans*
**N**	2	152,334.0 ± 4,406.7	49.8 ± 0.0	264.0 ± 9.9	130/409 (32)	M	*Rhizobium*
**O**	4	194,472.0 ± 8,712.4	49.0 ± 0.1	375.0 ± 18.9	322/426 (76)	M	*Sinorhizobium*
**P**	6	228,194.0 ± 26,162.1	65.7 ± 1.7	347.2 ± 49.7	79/767 (10)	S	*Caulobacter*
**Singleton**	28	67,533.4 ± 45,701.6	54.1 ± 14.1	102.3 ± 69.7	ND	N/A	N/A

^*a*^ P, podovirus; S, siphovirus; M, myovirus.

### Morphotypes demonstrate highly variable genome sizes

This set of sequences consisted of phages that were reported as myoviruses (*n* = 16). We found that the average genome size was 117,208 ± 69,543 bp; siphoviruses (*n* = 17) had an average genome size of 114,327 ± 87,204 bp; and podoviruses (*n* = 46) had an average genome size of 48,918 ± 15,217 bp. The 12 phages with unknown morphology had an average genome size of 66,935 ± 38,625 bp. Regardless of virion morphology, we observed an average GC% content of 52 ± 9%, which was highly dependent on the host genus. There were also relatively large genomes with unknown morphology (*n* = 22), as well as a single Leviviridae genome and a single Caudoviricetes genome.

### Genome cluster relationships occur at the host family level but not at the class level

**FIG 1:**
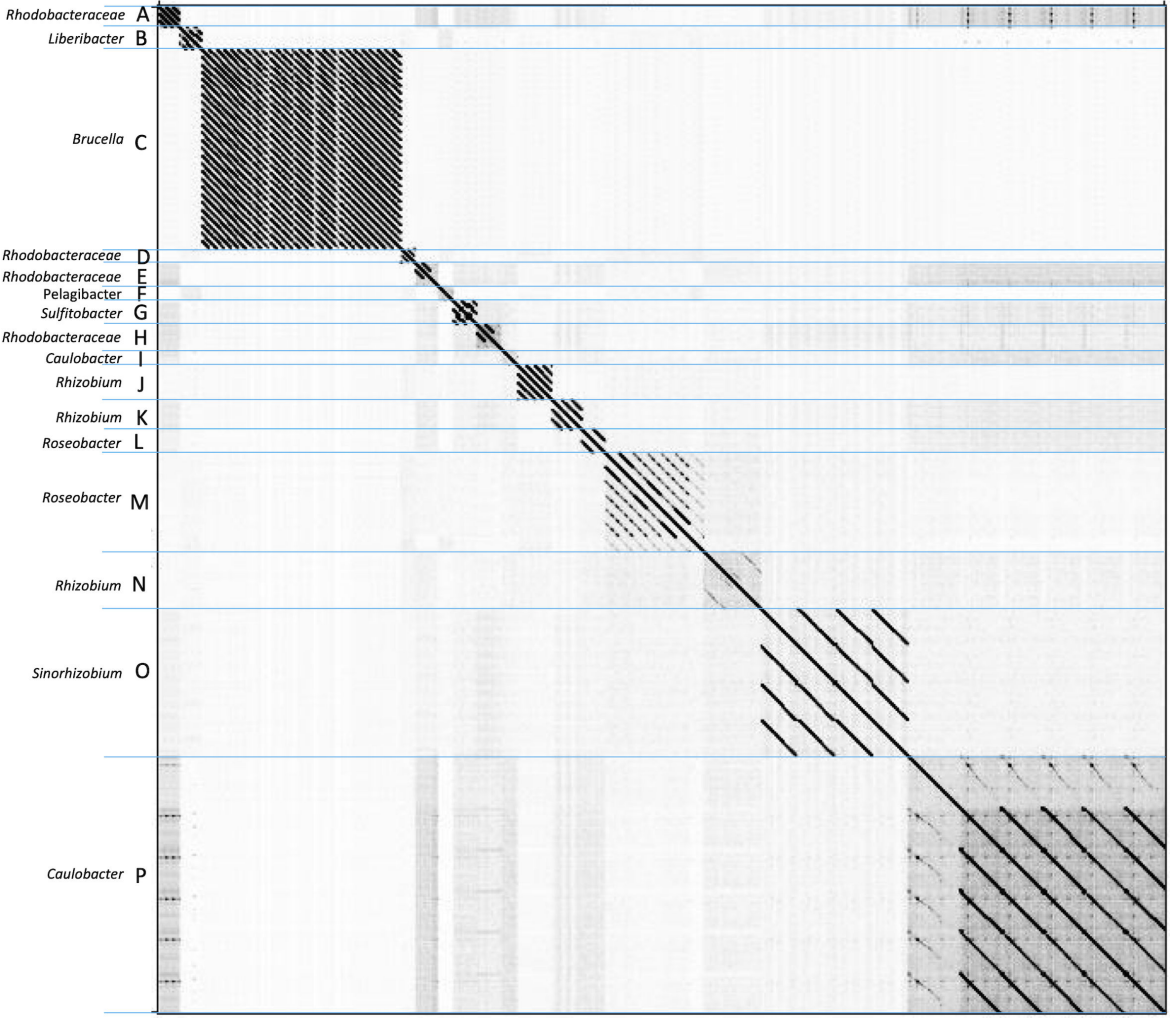
Dot plot generated from the whole genomes of phages that infect Alphaproteobacteria. Assigned phage clusters, together with the host bacteria that they infect, are labeled on the y-axis. Darker regions indicate more sequence identity, while blue lines indicate the boundaries of each phage cluster.

We created whole genome dot plots to characterize the sequence similarity between the genomes of these phages. Briefly, this method identifies regions where pairs of phages have identical sequence regions in either orientation. An advantage of dot plots over more typical phylogenetic analysis is that dot plots can detect homology in highly mosaic genomes that include historical sequence deletions, insertions, inversions, and rearrangements (46). Using this approach, we identified 16 phage clusters, 5 subclusters, and 28 singletons that we have named cluster A through cluster P (Fig. 1). We also identified a singleton phage that showed little or no similarity to any other known genome from phages that infect Alphaproteobacteria.

To verify cluster assignments, we aligned the same sequences that were used to construct the dot plots with Kalign prior to calculating the ANIs (see Materials and Methods for more details). We found that members of each cluster from the dot plot had an average ANI of at least 67%, but when comparing phages within a cluster, the lowest ANI value was 53%. In other words, we observed that the lower ANI threshold for phages that infect Alphaproteobacteria was ~53%. Genomes with an ANI less than this value were still included in our analysis but no longer had sufficient phylogenetic signal to be assigned to the same cluster as more closely related genomes. A final confirmation of cluster relationships was also ascertained by observing small deviations in average phage properties such as genome size, number of ORFs, and GC content (Table 1).

The *Brucella* phages, which comprise cluster C, had an average ANI value of 99.9%, which was the least diverse of any cluster that we observed in this study. In comparison, the most diverse set of sequences belonged to cluster M, which is composed of the genomes of phages infecting four bacterial genera: *Dinoroseobacter*, *Ruegeria*, *Sulfitobacter*, and *Roseovarius*. These phage genomes had an average ANI value of approximately 67% and reflected the pattern seen in Grose and Casjens’ study of the Enterobacteriaceae (19).

Singleton phages would be expected to have low ANI values when compared with any phage assigned to a cluster. The 28 singleton phages (27.5% of the phages included in this study) had average ANI values of ~40% and were not similar enough to constitute a cluster or to be assigned to a proper cluster. Interestingly, almost all bacterial genera in the class Alphaproteobacteria have phages that are classified as singletons. Since singletons have been observed at the species level for *Mycobacterium smegmatis* mc^2^155 (47), it was expected that we would have observed the same for a bacterial class. As more phages are discovered and assigned to the appropriate cluster over time, we posit that the percentage of singletons will decrease.

### Nucleotide dot plot analysis reveals subclusters in five clusters

Our analysis of the whole phage genome nucleotide dot plots revealed that some clusters are composed of a few phages that are more closely related to each other than others (Fig. S1). We grouped these phages as subclusters, following the precedent set by Grose and Casjens (19). We defined subclusters as phage sequences with ANI values greater than 65% when compared to other phages in the cluster. We identified evidence of subclusters within clusters B, E, H, M, and P. In cluster P, for example, subcluster P1 shared ANI values of greater than 75% when compared to other phages in the cluster. *Caulobacter* phage CcrColossus was identified as the sole member of subcluster P2. While having some degree of similarity to other phages in the cluster (between 51 and 56% ANI identity), its ANI was considerably less than the value of 75% that we observed when distinguishing between phages in subcluster P1.

### Amino acid dot plot analysis of the terminase large subunit protein confirms clusters

**FIG 2:**
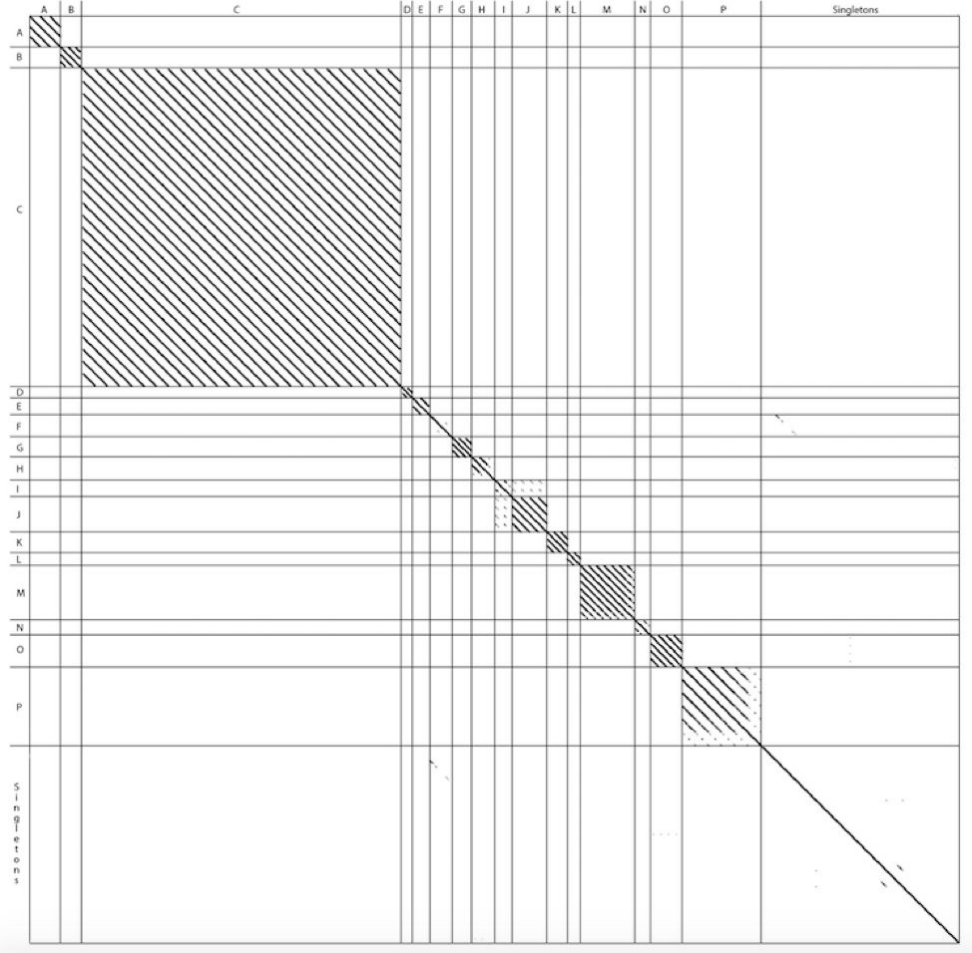
A dot plot generated from the amino acid sequence for the terminase large subunit protein from all clusters can closely reconstruct whole genome cluster assignments.

In addition to ANI, dot plots of amino acid sequences for well-chosen gene products have previously been shown to validate cluster assignments (19). We used this knowledge to determine the extent to which the clusters could be reconstructed based on the aligned amino acid sequences of the Terminase Large Subunit across the various phages. This analysis showed that the clusters could be accurately represented based on this single gene product (Fig. 2).

### The phylogenetic tree shows clades largely dependent on host bacterial species

**FIG 3:**
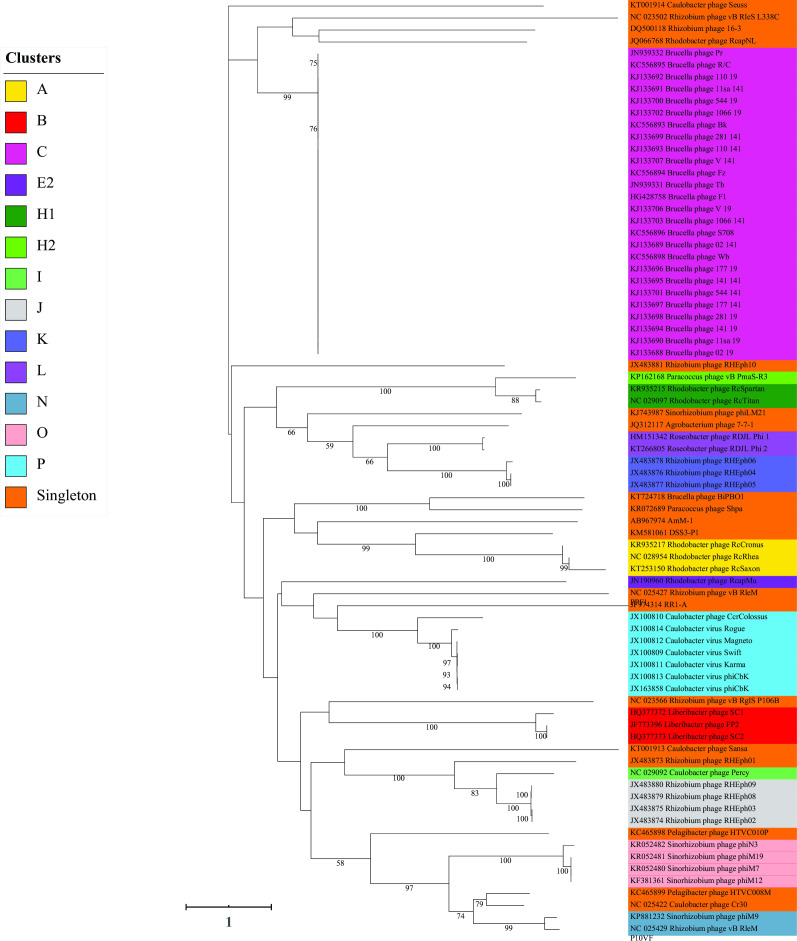
A maximum likelihood phylogenetic tree of amino acid sequences for the terminase large subunit protein from phages that infect Alphaproteobacteria, with 100 bootstrap replicates. The represented leaves are color-coded by cluster, and the scale bar represents the number of amino acid changes per site. A subset of the phages characterized in this work (79 of 103) are represented. Phages that are not present, including those in cluster M, lack an annotated terminase large subunit protein.

To gain a better understanding of the evolutionary relationships between these phage sequences, we reconstructed a phylogenetic tree based on the highly conserved large terminase subunit protein (Fig. 3). The results show that the sequences of phages isolated from the bacterial *Brucella* or *Liberibacter* genera tend to form well-defined monophyletic clades. In contrast, phage sequences collected from other bacterial genera (e.g., *Rhizobium*, *Rodobacter*, or *Sinorhizobium*) were observed to be polyphyletic and located across multiple clades of the phylogenetic tree. We also observed that a subset of phages isolated from the bacterial genus *Caulobacter* clustered together, while sequences from other phages isolated from the same genus were scattered throughout the tree. As expected, several phages isolated from a small subset of bacterial taxa (e.g., *Puniceispirillum*, *Agrobacterium*, or *Pelagibacter*) had insufficient sequence representation and were consequently placed as singletons in the tree. We observed that the larger phylogenetic clades tended to contain our assigned clusters, with singletons intermixed among the well-defined clusters.

### Phage superclusters are confirmed using gene phamilies

We next wanted to identify any detectable phage superclusters by predicting gene “phamilies” or “phams,” which represent orthologous proteins. A supercluster is a group of phage sequences that are not similar enough to constitute a cluster but share conserved structural and functional genes. Superclusters identified by Grose and Casjens (19) were named after phages that were first isolated and studied. In general, faint lines in the whole genome nucleotide or amino acid dot plots, or other regions having less than 50% similarity across the whole genome or proteome, can indicate distant evolutionary relationships. We confirmed the presence of these supercluster relationships by analyzing phage gene phamilies identified in Phamerator. We observed that some phams were unique to an individual cluster, whereas others were shared among multiple clusters. We also found that some phams had similar gene synteny across different phages and were consequently grouped as a supercluster. Specifically, for a phage genome to be considered part of a supercluster, the genes need to be similar in content and in synteny.

We observed these attributes in a subset of the phage genomes, which shared gene synteny with phage T4 and was consequently identified as the T4-like supercluster. For example, the *Sinorhizobium meliloti*-infecting phages that belong to cluster O in this study have previously been determined to be members of the Enterobacteriaceae phage T4-like supercluster (48 – 50). An analysis of Phamerator genome maps added the cluster N phage, *Pelagibacter* phage HTVC008M, and *Caulobacter* phage Cr30 to the T4-like supercluster (51). Notably, all of these phages have a section of their genomes that contains the terminase protein, which is followed by tail sheath stabilizer proteins, viral neck proteins, tail sheath and tail tube proteins, portal vertex proteins, proteins involved in DNA synthesis, and major capsid proteins, in the same order. Notably, phage clusters N and O share 22 phams, 17 of which are also shared with phages Cr30 and HTVC008M.

The three phages belonging to cluster E and the singleton *Rhizobium* phage RR1-B constituted a potential Mu-like supercluster. Genes that are shared with phage Mu encode a putative nuclease inhibitor, a major head subunit protein, and a terminase. Whereas 16 of the 57 phams (28.07%) in phage RcapMu are shared with phage RR1-B, phage Rcap-Mu shares only 11 phams (20%) with *Rhodobacter* phage RC1 and *Rhodovulum* phage RS1. However, no similarity was demonstrated with any of the other phage genomes in the supercluster. These genomes share regions of considerable synteny with the terminase protein, followed by endolysins and autolysins, helix-turn-helix domains containing transcriptional regulators, a Mu-type integrase, virion morphogenesis protein, major capsid protein, and tape measure protein.

A third supercluster, the T3/T7-like supercluster, is formed by clusters F, J, and I and the singleton phages *Mesorhizobium* phage vB_MloP_Lo5R7ANS and *Rhizobium* phage RHEph01. Cluster F and *Mesozrhizobium* phage vB_MloP_Lo5R7ANS have nine phams also found in T3, cluster J has four shared phams with T3, and cluster I shares three phams with T3. Representative phams shared by members of this supercluster include an RNA polymerase, a bacteriophage head-to-tail connecting protein, a family A polymerase, a replicative DNA helicase, a phage endonuclease, a major capsid protein, and a peptidoglycan recognition protein. These genes were previously identified by Grose and Casjens as being highly conserved genes in the T7 supercluster (17).

### Conserved gene products further define phage relationships

We used gene phamilies, generated by Phamerator and proteinOrtho (Table S2), having an *E*-value of less than 1 × 10^−50^, and 32.5% identity, to further study the relationships between phage clusters. Of the 4,676 phams, 3,813 (81.6%) had no known function, and 3,495 (74.7%) were orphams (i.e., phams that only contained a single member). Pope et al. (18) previously showed that singleton phages and subclusters that contain a single phage tend to have a high orpham percentage, whereas multi-phage subclusters containing multiple representative phages have a low orpham percentage. Not surprisingly, the phages in our study followed the same pattern.

**FIG 4:**
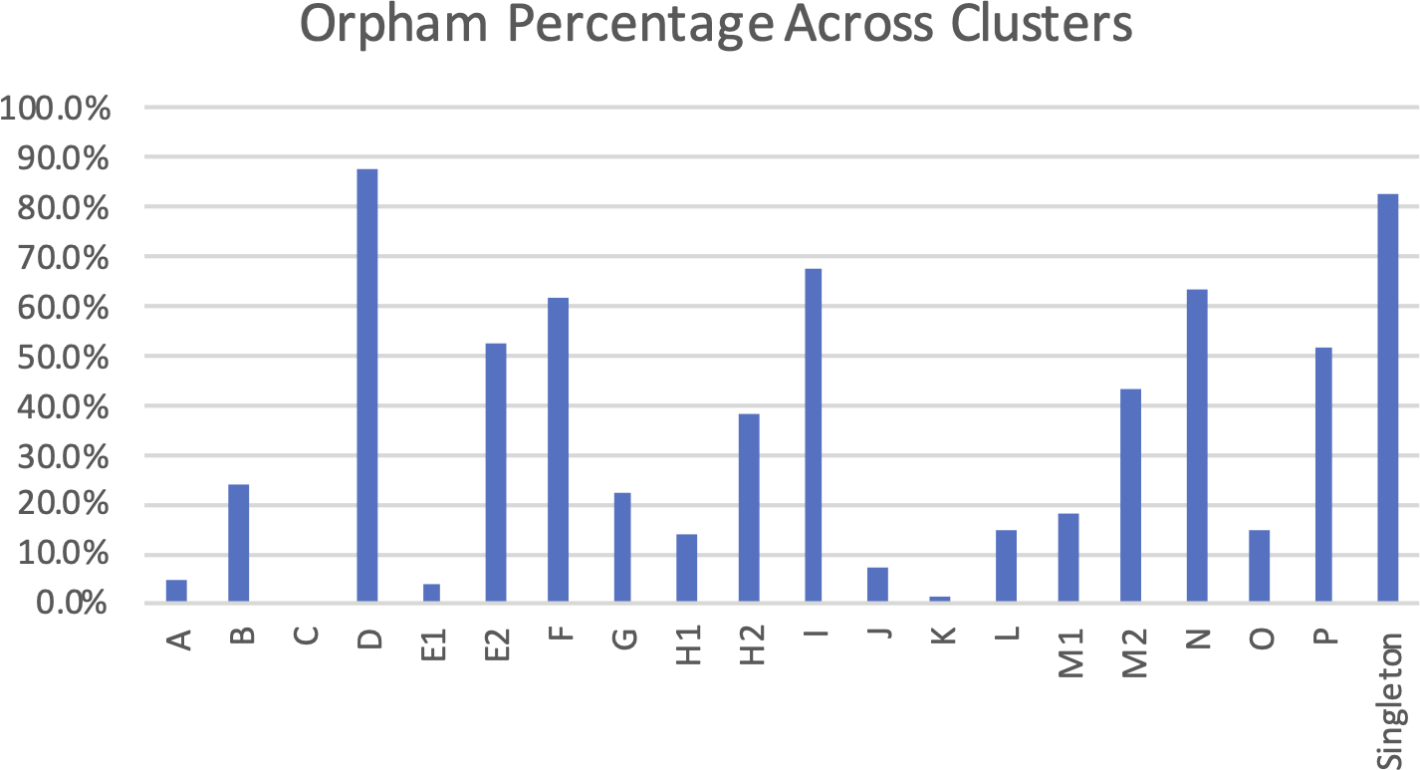
Diversity in phage orphams (phams that only contained a single member) percentages across assigned clusters. The number of gene orphams assigned across all phages and clusters included in the current study shows a large range of diversity between clusters.

We observed that the majority of the singleton phages had an orpham percentage of 60% or greater. At the individual phage level, we observed that the orpham percentages of the phages that have been assigned to clusters vary greatly within each cluster. Clusters that contain highly related phages, like clusters B or G, have orpham percentages of less than 1%. Clusters that contained less-elated phages, like clusters C or J, had between 20% and 60% orphams (Fig. 4). We found a similar trend after quantifying the percentage of orphams across all phages assigned to each cluster. The Singleton cluster had 82.5% orphams across all its members, which was only surpassed by cluster D at 87.5%. The clusters with the lowest orpham percentages were C (0%), K (1.2%), E1 (3.5%), and A (4.3%). Based on the metadata describing the bacterial hosts that these phages were isolated from, these differences are mostly attributable to the diversity of the host range, with likely contributions from genome length, genome organization, and other factors.

### Cluster Isolation Index reveals cluster diversity

**FIG 5:**
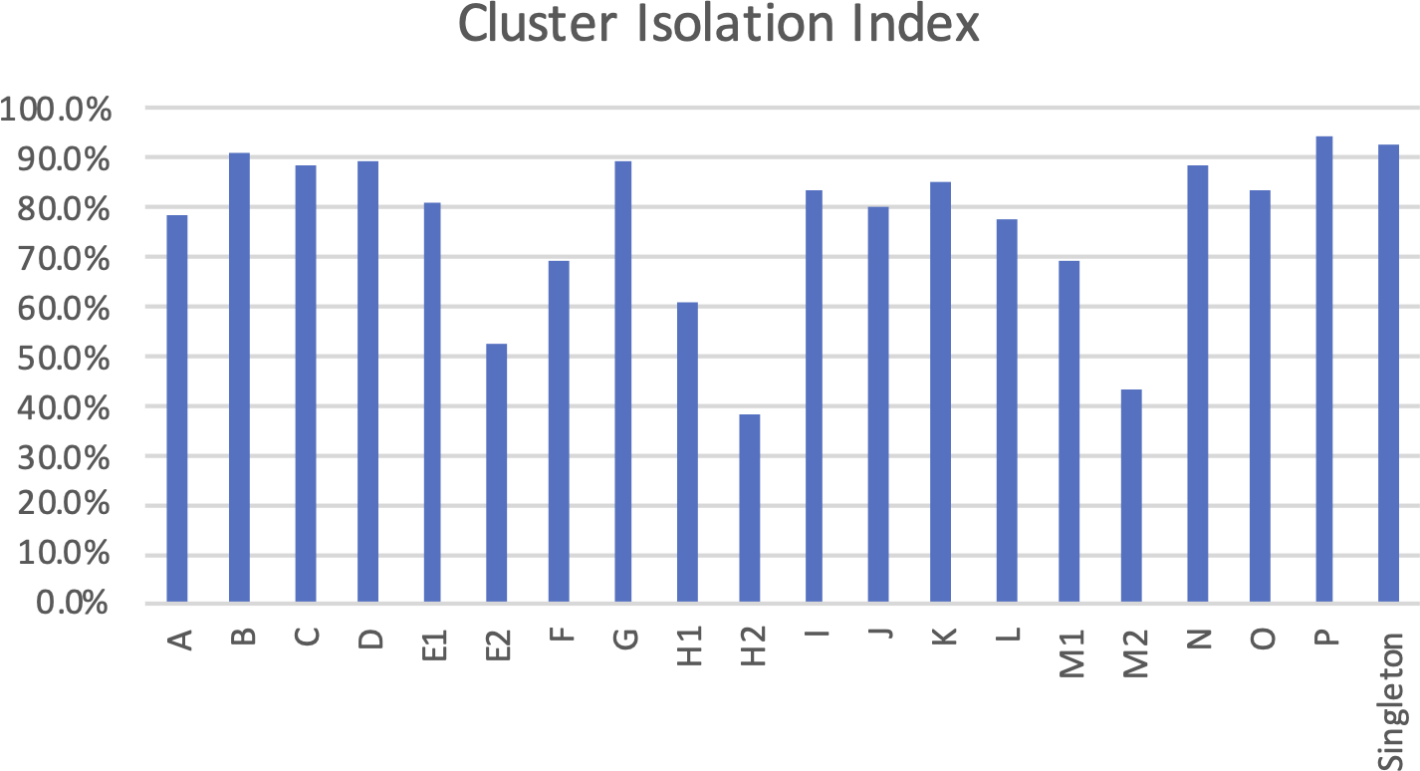
CII distribution. The percentage of non-redundant phams that are unique to each cluster.

The relationships between phage clusters can be quantified using the Cluster Isolation Index (CII), as shown by Pope et al. (52). The CII is the percentage of phams that are found within a cluster that are not found in any other phage genomes. A high CII is interpreted to mean that the phages in that cluster are not highly related to any other phage. We calculated the CII based on the number of non-redundant phams in our assigned clusters (Fig. 5). This analysis revealed an interesting distribution of unique phams by cluster assignment. On the lower end of the distribution, we found that 38% of phams were unique to cluster H2, while at the higher end, we saw 95% of phams that were unique to cluster P. We noticed that clusters consisting of phages with larger genomes tended to have a larger CII. This is not surprising since larger phage genomes tend to include accessory proteins that can become highly specialized and therefore only present in a well-established cluster.

### Cluster A

The genomes of the three cluster A phages, reported by Bollivar et al. (53), are extremely similar. Shared protein ortholog groups among members of this cluster include a terminase, major capsid protein, tail proteins, a DNA primase, a head-to-tail joining protein, a tail assembly chaperone, and a portal protein.

### Cluster B

The phages in this cluster are not closely related to any of the other clusters or singletons in this study, with only two of the cluster B phams (helicase and family A DNA polymerase) present in phages of cluster M: *Ruegeria* phage DS33-P1 and *Sinorhizobium* phage phiM5. We report two subclusters in cluster B: subcluster B1, which contains *Liberibacter* phage SC2 and *Liberibacter* phage FP1, and subcluster B2, which contains *Liberibacter* phage SC1. All three phages share multiple protein ortholog groups of enzymes involved in DNA replication, including a DNA/RNA helicase, a NAD-dependent DNA ligase, and a family A DNA polymerase, a bifunctional DNA primase/polymerase. As expected, phages in subcluster B1 (SC1 and FP1) are very closely related, with an ANI value of 99.93%, whereas SC1 has an ANI value of 76% when compared to the other two phages in the cluster. The fact that SCI and SC2 have been assigned to different subclusters is notable. While early coding regions had 93% overall sequence identity, there were unique genes in the late-coding regions (54) that would account for the difference in cluster assignment. While these genomes shared identical putative primases, endonucleases, DNA polymerases, helicases, and DNA ligases, it was the structural genes that differed in sequence identity.

### Cluster C

We found 26 podoviruses in cluster C, which infect *Brucella* spp. These zoonotic bacteria cause brucellosis in mammals, including humans. We observed that the genomes for these phages, like those in cluster A, are highly similar. Only seven of the cluster C phams are present in any other genome included in this study. Putative protein ortholog groups shared by this cluster include those involved in DNA metabolism and editing. Of these, there is a putative HNH endonuclease, a putative helicase, a family B DNA polymerase, a bifunctional DNA primase/polymerase, a methyltransferase, and a bacterial DNA A-like protein. These phages also share a few important enzymes, such as endolysin, pepidoglycan hydrolase, and a terminase protein. There are two interesting bacterial proteins encoded by these genomes: a chromosome segregation ATPase also found in *Mycoplasma* bacteria and a helix-turn-helix domain similar to the *Salmonella* Hin protein (a recombinase). We observed that these phages also share a few phams (an acetyltransferase, a chaperone of endosialidase, a lysozyme, an and HNH endonuclease) with the cluster G phage.

### Cluster D

The cluster D phages are more distantly related, as evidenced by only having 21 of the 62 (34%) phams present in both phages belonging to this cluster. These phages share protein ortholog groups that include a thymidylate synthase, a phage endonuclease, a Class II ribonucleotide reductase, a T-7-like phage DNA polymerase, a phage-associated DNA polymerase, and a putative phage tail fiber protein.

### Cluster E

Cluster E contains two subclusters of phages that also infect bacteria in the Rhodobacteriaceae family: E1 has two closely related phages, *Rhodobacter* phage RC1 and *Rhodovulum* phage RS1, and E2 has a single phage, *Rhodobacter* phage RcapMu. Unlike the phages belonging to cluster D, these are siphophages and infect different species of the Rhodobacteriaceae family. Our findings, combined with prior results, suggest that the cluster E phages likely belong to a Mu-like supercluster. These phages share protein ortholog groups that include a phage-related lysozyme, a tape measure protein, tail proteins, a major head subunit, a DNA-binding protein, a phage repressor, a Mu DNA-binding protein, and an integrase protein.

### Cluster F

Many of the phams identified in cluster F are shared with other phages that infect Alphaproteobacteria, including clusters C, D, I, and J, as well as the singleton phage *Mesorhizobium* vB_MloP_5R7ANS and *Rhizobium* phage RHEph01. Some of the protein ortholog groups for this cluster are structural proteins such as a head-to-tail connecting protein, a capsid protein, and a tail fiber protein, as well as other important enzymes like a DNA family A polymerase, a DNA primase, a DNA family B maturase, and an exonuclease.

### Cluster G

Cluster G contains three highly related phages: *Sulfitobacter* phage phiCB2047-A, *Sulfitobacter* phage pCB2047-C, and *Sulfitobacter* phage NYA-2014a. The protein ortholog groups shared in this cluster included genes encoding enzymes such as an acetyltransferase, a lysozyme activator protein, an HNH endonuclease, an integrase, and a terminase enzyme.

### Cluster H

Cluster H contains three phages that comprise two subclusters. Subcluster H1 contains *Rhodobacter* phage RcTitan and *Rhodobacter* phage RcSpartan, and the second subcluster contains *Paracoccus* phage vB_PmaS_IMEP1. Many of the conserved protein ortholog groups for this cluster do not have a known function, but there are some conserved enzymes such as a terminase protein, an exonuclease protein, a DEAD-box helicase, and a replicative clamp protein, as well as scaffold and portal proteins.

### Cluster I

This cluster contains two podoviruses that infect *Caulobacter* spp.: *Caulobacter* phage Cd1 and Percy. They share protein ortholog groups that include most of their structural genes (head-to-tail joining protein, tail tubular protein A and B, major capsid protein, and internal virion protein) and enzymes for DNA/RNA metabolism (family A polymerase, DNA ligase, RNA polymerase, and DNA polymerase). The cluster I phages are members of the T3/T7-like supercluster, sharing seven putative structural or replication proteins with the other members of the supercluster.

### Cluster J

These phages also shared a muramidase/lysozyme similar to that found in lambda phage, a T7-like tail fiber protein, and a spore core-lytic enzyme from *B. subtilis*, which is used to degrade the peptidoglycan cortex during spore germination (55). The phages in this cluster shared phams including DNA helicase, family A polymerase, recombination endonuclease VII, a DNA-dependent RNA polymerase, a head-to-tail joining protein, and a major capsid protein with the following singleton phages in clusters F and I: *Mesorhizobium* phage vB_MloP and *Rhizobium* phage RHEph01, all of which belong to the T3/T7-like supercluster.

### Cluster K

The shared protein ortholog groups include genes encoding DNA/RNA metabolism proteins, a DNA polymerase, a helicase, and an RNaseH, as well as structural enzymes like a terminase large subunit, a head morphogenesis protein, and a capsid protein. These phages also contained two enzymes involved in tetrahydrobiopterin synthesis: GTP cyclohydrolase, which is involved in tetrahydrofolate synthesis, and 6-pyruvoyl-tetrahydropterin synthase, which catalyzes the second step of tetrahydrobiopterin synthesis, the conversion of dihydroneopterin to 6-pyruvyltetrahydropterin (56). They also contained the queuosine biosynthesis protein QueC, which is involved in purine metabolism, and a radical SAM superfamily protein, an iron-sulfur-containing enzyme that catalyzes the cleavage of S-adenosyl-L-methionine (57). The presence of these bacterial proteins provides further evidence that cluster K contains temperate phages (58).

### Cluster L

Cluster L is made up of two closely related phages: *Roseobacter* phage RDJLphi1 and *Roseobacter* phage RDJLphi2. These siphoviruses, first reported on by Zhang and Jiao, infect the marine bacterial taxon *Roseobacter denitrificans* OCh114 (59). The conserved phams include proteins involved in DNA synthesis such as a family A DNA polymerase, a helicase, an integrate protein, a transcriptional regulator, an endonuclease protein, as well as a DNA repair enzyme—a pyrmidine dimer DNA glycosylase. These phages also share many different protein ortholog groups, including a terminase enzyme large subunit, a putative phage cell wall peptidase, a dihydrofolate reductase, a thymidylate synthase, a nucleoside, a head morphogenesis protein, a phage integrase, a tail tape measure protein, an HD superfamily hydrolase, and a deoxycytidylate deaminase.

### Cluster M

We found that cluster M contained seven podoviruses that infect marine members of the Rhodobacteraceae family. Our analysis further divided this cluster into two subclusters. Subcluster M1 contains *Roseovarius* spp. 217 phage 1, *Roseovarius* Plymouth Podovirus 1, Roseophage DSS3p2, Roseophage EE36P1, *Dinoroseobacter* phage DFL12phi1 (formerly named Roseophage vB_DshP-R1), and *Dinoroseobacter* phage vBDshPR2C, while subcluster M2 has only one phage, *Sulfitobacter* phage phiCB2047-B. These phages are N4-like podoviruses and show some homology with phages that infect Achromobacter JWDelta (60, 61). The genes in this cluster contain substantially more sequence variation than many of the other clusters. All N4-like phages share 14 core genes: RNAP1 and RNAP2 transcriptional controls, the vWFA domain, DNA polymerase, SSB, vRNAP (involved in DNA metabolism), structural proteins including terminase, major capsid, and portal proteins, and five genes of unknown function (62). Other shared protein ortholog groups include DNA helicase, a family A DNA polymerase, a RNA polymerase subunit, a ribonucleoside diphosphate reductase, and a rIIB-like protein.

### Cluster N

We observed two podoviruses that comprise cluster N, which infect two different species of the Rhizobiaceae family, *Rhizobium* phage vB_RleM_P10VF and *Sinorhizobium* phage phiM9. As originally reported by Johnson et al., these phages belong to a T4-like supercluster, though they have less synteny with T4 than they do with other members of the T4-like supercluster (49). In addition to the shared structural and DNA metabolism protein orthologs, these phages all contain a peptidoglycan recognition protein, a bifunctional gluaredoxin/ribonuceloside diphosphate reductase, an FAD-dependent oxidoreductase, a chaperone of endosialidase, and a trypsin-like serine protease (49). They contain bacterial proteins such as a KaiC domain-containing protein that regulates circadian rhythms in cyanobacteria, a ComE operon protein, a PhoH protein from *Escherichia coli*, and a conjugal transfer fertility inhibition protein, a ubiquitin activating protein found in eukaryotes, and a tropomyosin-like chromosome segregation protein.

### Cluster O

We found that cluster O contains four myoviruses that all infect the bacteria *Sinorhizobium meliloti*. These phages have been identified multiple times as members of a T4-like supercluster (48 – 50). We observed that the genes in these phage genomes are fairly similar, with 322 of the 426 (~76%) cluster O phams present across all members of the cluster and showing remarkable synteny. The shared protein ortholog groups for this cluster include enzymes used in DNA metabolism and repair such as a RecD-like helicase, a DNA primase, a clamp loader small subunit, a DNA polymerase, an RNA polymerase sigma factor, a methanogen-like thymidylate synthase, a nucleotidyltransferase, an exonuclease, an Mre11 nuclease (a protein used by *E. coli* for double-stranded break repair), a RecA protein, a class II ribonucleotide reductase, and a dUTPase protein. These phages also share structural proteins and enzymes such as a major head protein, a prohead core protease, a tail tube and tail sheath protein, a terminase protein, a neck protein, a head completion protein, as well as other enzymes such as: a nicotinamide phosphoribosyl transferase, a glycosyltransferase, a GrxA family glutaredoxin, a metal-dependent phosphohydrolaseprotein, a chitinase, an Appr-1′-p processing enzyme, a dihydrofolate reductase, a NAD synthetaseprotein, a PnuC nicotinamide riboside transporter, a, a peptidyl-tRNA hydrolase, a tRNAnucleotidyltransferase-like protein, rare lipoprotein A, a calcineurin-like phosphoesterase, and PPAT (a bacterial enzyme involved in CoA biosynthesis, and S-adenosylmethionine decarboxylase (bacterial spermidine synthesis). Further evidence that these phages belong to a T4-like supercluster of phages can be found in analyses of their gene products. All of these phages contain a T4-like baseplate protein, a T4-like RNA ligase, a UvsY protein (a T4-like protein that is used in recombination-dependent DNA synthesis), and a regA translational repressor that is used by T4 to control early gene expression (63).

### Cluster P

We designated cluster P as containing six siphoviruses that infect *Caulobacter crescentus*, a freshwater bacterium. This cluster contains two subclusters: P1 contains *Caulobacter* phages CcrKarma, CcrMagneto, CcrSwift, CcrRogue, and phiCbK, and P2 contains a single phage, *Caulobacter* phage CcrColossus. The relationship between these six phages was first reported by Gill et al. when they classified these phages as a phiCbK-like group and reported that the shared conserved orthologs consist of structural proteins involved in DNA metabolism (64). In addition to these conserved proteins, we identified other shared protein ortholog groups, including a concavalin A-like lectin/glucanase, a PhoH protein, a putative cell wall peptidase, a lysozyme, a ribonucleoside-diphosphate reductase, a thymidylate synthase, a deoxynucleoside monophosphate kinase, a site-specific DNA methylase, an integrase, a nicotinamide phosphoribosyl transferase, and a nicotinamide/nicotinate mononucleotide adenylyltransferase.

### Clustering somewhat reflects diversity in packaging strategies

We wanted to predict phage packaging strategies for these phages, where possible, using an existing phylogenetic-based analytical method. Briefly, we used this method to compare the terminase amino acid sequence from our phages that infect Alphaproteobacteria against a collection of representative terminase proteins that use a variety of packaging mechanisms, including 5′COS, headful, direct terminal repeat (DTR), 3′COS, and host ends. We observed that a subset of terminase sequences from our phages shared a well-defined ancestry with terminases that have a characterized packaging strategy, although this finding was not consistent across all of our identified clusters (Fig. S2).

We found that the cluster E2 phage likely uses a host ends/D32 strategy, while clusters K and L both use a headful/Sf6 mechanism with high phylogenetic confidence. We also saw that the singleton phages displayed high phylogenetic evidence of using a variety of mechanisms, including 3′Cos/HK97, short DTR, and headful Sf6.

In contrast, we observed several phages that displayed less confidence in their packaging method, including those belonging to clusters A and B (5′Cos/lambda), clusters H1 and H2 (headful/P22), cluster I (headful/P22 and short DTR), clusters N and O (headful/T4), and singletons (headful/T4 and 5′Cos/Lambda). The rest of the terminase sequences from the Alphaproteobacteria phage clusters in our analysis had either distant ancestry or mixed ancestry with the characterized phages. As such, we were less confident in predicting the potential packaging mechanism for these phages.

## DISCUSSION

The aim of our analysis was to better characterize the sequence diversity that exists among phages that infect Alphaproteobacteria. Our analysis quantified the genome size, GC%, core gene set, number of ORFs, protein ortholog groups, and phage gene orthologs. Overall, we identified multiple clusters and subclusters of phages that shared similar attributes and orthologs, as well as a relatively large subset of singleton sequences that did not strongly cluster with other phage genomes.

This study is the first to compare phages infecting the Alphaproteobacteria class. It appears from our single study of only 103 genomes that phages capable of infecting a bacterial class have little similarity in overall genome architecture; however, genomes are more likely to cluster within phages that infect a bacterial family. These 103 genomes, like other phage genomes that have been studied (18, 19, 21), cluster strongly with the bacterial host family, phage morphotype, and genome size. We found that genome clusters were composed of only phages that infected a specific host.

The three cluster A phages have been previously characterized as having high similarity (53). Interestingly, during stationary growth, some members of *R. capsulatus* releases a novel phage-like horizontal gene transfer agent, RcGTA (65). *Rhodobacter capsulatus* is a purple non-sulfur bacterium that is capable of fixing nitrogen. It has a gene transfer agent that has the appearance of a phage and which, during stationary growth, is capable of passing along 4 kb segments of bacterial DNA. In addition to the GTA-like tail fiber protein reported by Bollivar et al., our analysis identified a GTA TIM-barrel-like domain and a plasmid replication initiator protein (41).

Cluster B contains three podophages that infect the intracellular plant pathogen *Candidatus Liberibacter* spp. The availability of these phage genome sequences is quite remarkable since their bacterial host, *Candidatus L. asiaticus*, is yet to be continuously cultivated in the laboratory (66). These phage genomes are *Liberibacter* phage SC1, *Liberibacter* phage SC2, and *Liberibacter* phage FP2. The genomes of phages SC1 and SC2 were observed as prophage excision plasmids in host bacteria that were infecting plant material (54).

The 26 cluster C phages infect only *Brucella* spp., which was expected since *Brucella* spp. are the only members of the bacterial Brucellaceae family. Although not specifically included in the study that described three subclasses within the Alphaproteobacteria (3), *Brucella* spp. appear to be more closely aligned with the Caulobacteridae subclass. By contrast, phages that infect *Rhodovulum* and *Rhodobacter*, two closely related genera of Rhodobacteriaceae, were members of the same genome cluster (cluster E). This pattern was previously observed in the analysis of 337 Enterobacteriaceae phage genomes studied by Grose and Casjens, with some clusters sharing phages that infect various genera and others being specific for one host (19). It is unknown if this is due to phage lifestyle (the type of phage), overlapping ecologies of the bacterial hosts, or sampling biases. The phages in cluster C shared 54 of the 59 (92%) phams, which is a similar value to that reported by others (67, 68). Our data support those of Tevdoradze et al., who reported similar pairwise percent identity and synteny among 22 phages that infect Brucella (67). They recognized that a lack of diversity among these genomes, despite disparate places of phage isolation, suggested little to no recombination and that the genes may have been adapted and undergone selection to improve infection with *Brucella* spp. Our analysis confirms this lack of diversity among the genomes of the cluster and additionally finds very little similarity with any other phage genome in the cluster. Whole genome phylogenetic analysis of six phages that infect Brucella by Farlow et al. revealed three groups that corresponded to three host range phenotypes (68). Such resolution was not shown by our analysis. This is likely due to the fact that the recognized definition of a cluster is very broad, whereas these group assignments were made based on very minor differences between the various genomes. In addition, our phylogenetic analysis focused on the amino acid sequence of the large terminase subunit, which provides less resolution than a tree that is reconstructed from the whole genome nucleotide sequence. However, generating a phylogenetic tree of all nucleotides or comprehensive protein sequences across these phages would likely prove intractable given the diversity of sequence, presence or absence of genes, and gene synteny.

Cluster D only contains two podoviruses that infect different species in the family Rhodobacteriaceae: *Celeribacter* phage 12053L and Roseophage SI01. The latter of these phages infects the marine heterotrophic bacterium Roseobacter SI067 (69). Of the five newly identified strains of Roseophage SIO1, one strain (strain sbrsio67) was found to also infect *Roseobacter* GAI-101 (70). The strains vary in three genome regions: a *thyX* gene, a gene for phosphate metabolism, and various structural genes (70). The similarity between these two phages was first discovered by Kang and colleagues in the genome announcement for *Celeribacter* phage 12053L (71).

Cluster E phages primarily infect *Rhodobacter*, a purple photosynthesizing bacteria that is found in seawater, and *Rhodovulum*, another marine photoheterotroph. Our observation that these two phages contain genes encoding an integrase as well as other prophage-related proteins leads us to believe that they are temperate phages. Upon its discovery, *Rhodobacter* phage RcapMu was identified as a temperate phage capable of performing inducible transposition in a manner similar to that of Enterobacteria phage Mu (72). Homologs of structural proteins from *Pseudomonas* and *Burkholderia* phages were also identified in RcapMu, including the head assembly, capsid, and tail proteins (72).

The three phages assigned to cluster G are temperate podoviruses first isolated and sequenced by Ankrah et al. (73). Phages phiCB2047-C and phiCB2047-A were found to be extremely similar, differing only in an approximately 2,000-bp region containing five hypothetical proteins: phiCB2047-C and a RusA-like endodeoxyribonuclease in phiCB2047-A (73). These phages also shared bacterial proteins, such as a hemolysin XhlA protein, a flagellar rod assembly protein that included a muramidase, and a HipB protein that regulates multiple promoters in *E. coli* (74). Our analysis agrees with Ankrah et al. that these phages lack homologs of enzymes involved in DNA synthesis (73).

Bollivar et al. found that the phages in cluster H share some homology over approximately 35% of their genome with various phages capable of infecting *Salmonella, Pseudomonas,* and *Burkholderia* (41). In addition, these phages share some phams with the other *Rhodobacter*-infecting cluster A phages, including portions of the *Rhodobacter capsulatus* gene transfer agent, as well as a cell-wall peptidase and three hypothetical proteins of unknown function.

The differences between the phages assigned to cluster I, as first reported by Lerma et al., mostly consist of hypothetical proteins of unknown function (75). Interestingly, the only structural genes not shared by these two phages are those responsible for the tail fiber proteins, with each phage having a distinct gene.

Four podophages infecting rhizobia comprise cluster J. The *Rhizobium* phage RHEph02, *Rhizobium* phage RHEph03, *Rhizobium* phage RHEph08, and *Rhizobium* phage RHEph09 were reported as lytic phages that infect *Rhizobium etli* by Santamaria et al. (58). Rhizobia are a well-studied group of bacteria due to their relationship with the Leguminosae family of plants. *Rhizobium* may have co-evolved with the plants to form root nodules that fix nitrogen, helping the plants grow in nitrogen-poor environments (76). *Rhizobium*-infecting bacteriophages play a crucial role in this symbiosis, as bacteriophage-resistant *Rhizobium* species are better able to form root nodules than other less resistant species or strains (77). These phages were discovered along with the cluster K phages and the singleton phages *Rhizobium* phage RHEph01 and *Rhizobium* phage RHEph10. Santamaria et al. divided these phages into four groups, all of which are consistent with our cluster assignments (58).

The members of cluster L have been somewhat characterized previously. Specifically, Huang et al. reported that RDJLphi1 contained homologs of the *Rhodobacter capsulatus* gene transfer agent, which consisted of a cell wall peptidase of the NlpC/P60 superfamily and a rhamnosyl transferase (78). Our analysis also found these homologs in the genome of RDJLphi2. In addition to these *Rhodobacter capsulatus* homologs, these phages contain three bacteria-specific proteins: a nodulation protein A (from rhizobia), a ribonucleotide reductase and pyruvate formate lyase, and an *E. coli* protein involved in anaerobic glycolysis (79), and a ComE operon protein (*B. subtilis*), which is involved in DNA uptake during transformation. These phages also share phams with multiple clusters, singleton phages (clusters A, B, C, K, and M), *Agrobacterium* phage 7-7-1, *Paracoccus* phage vB_PmaS-Imep1, *Ruegeria* phage DSS3-P1, and *Sinorhizobium* phage phiM5.

Prior studies have endeavored to characterize the cluster M phages. Zhao et al. first reported the presence of genes for thioredoxin and ribonucleotide reductase that show significant homology to those found in *Roseobacter* (80). In addition to these genes, a few of these phages contain bacterial proteins. For example, all seven phages contain a pyruvate formate lyase gene; three of them have a ribosomal protein; four have an O-methyltransferase that is involved in bacterial antibiotic production; and four of them have a *B. subtilis* ComE operon protein, and two of them have a *B. subtilis* spore core lytic enzyme.

Prior work has shown that phage taxonomy and their receptors play a major role in host range specificity (81). As such, we are not surprised that some of the diverse phages in our analysis are capable of infecting the same bacterial host yet have been assigned to different clusters (82 – 84). We expect that the classifications identified in this work will change as the number of genomes from phages that infect Alphaproteobacteria increases. In particular, we believe that a subset of the singleton genomes will form clusters with the genomes of phages that have not yet been sequenced but that exist in nature. We also assume that the membership in our clusters will increase, with a high likelihood that subclusters for our clusters will need to be defined in the future.

### Conclusions

In summary, we identified phages belonging to 16 clusters, 6 subclusters, and multiple singletons from our comprehensive set of public genome records. The host range, genome size, nucleotide sequence, core gene set, number of ORFs, number of protein ortholog groups, and other attributes that we observed among these phages are relatively similar within each cluster and more diverse among the superset of phages. More in-depth analysis revealed that phages belonging to the same cluster or subcluster were able to infect a subset of bacterial taxa within the Alphaproteobacteria class. Our proposed cluster assignments and packaging mechanisms may shed additional light on observed phenotypes among members of the same cluster and will help in future classification efforts of these phages.

## SUPPLEMENTAL MATERIAL

Supplemental Figure Legends spectrum.02827-23-s0001.docx Additional supplemental file for only the legends.

Fig. S1 spectrum.02827-23-s0002.tiff Nucleotide sequence dot plots from members of three separate clusters showing the presence of subclusters in clusters B (upper-left panel), M1 (upper-right panel), and P (bottom panel).

Fig. S2 spectrum.02827-23-s0003.pdf Phylogenetic tree of the amino acid sequences from the terminase protein of phages that infect alphaproteobacteria and terminase sequences from phages with well-characterized packaging mechanisms. Scale bar represents % nucleotide diversity.

Table S1 spectrum.02827-23-s0004.xlsx Names and identifiers of all strains used in this study.

Table S2 spectrum.02827-23-s0005.xlsx List of predicted protein ortholog groups computed for this study.

ASM does not own the copyrights to Supplemental Material that may be linked to, or accessed through, an article. The authors have granted ASM a non-exclusive, world-wide license to publish the Supplemental Material files. Please contact the corresponding author directly for reuse.
