# Supplementary material for: Diversity and conservation of the genome architecture of phages infecting the Alphaproteobacteria
Source: Microbiol Spectr. 2023 Nov 22;12(1):e02827-23. doi: 10.1128/spectrum.02827-23 (PMC10783043; doi:10.1128/spectrum.02827-23)
Supplement: Supplemental Figure Legends — Additional supplemental file for only the legends. [file spectrum.02827-23-s0001.docx]

**Supplemental Material**

**Fig S1**: Nucleotide sequence dot plots from members of three separate clusters showing the presence of subclusters in clusters B (upper-left panel), M1 (upper-right panel), and P (bottom panel).

**Figure S2**: Phylogenetic tree of the amino acid sequences from the terminase protein of phages that infect alphaproteobacteria and terminase sequences from phage with well-characterized packaging mechanisms. Scale bar represents % nucleotide diversity.
