# Supplementary figures and images for: Diversity and conservation of the genome architecture of phages infecting the Alphaproteobacteria
Source: Microbiol Spectr. 2023 Nov 22;12(1):e02827-23. doi: 10.1128/spectrum.02827-23 (PMC10783043; doi:10.1128/spectrum.02827-23)

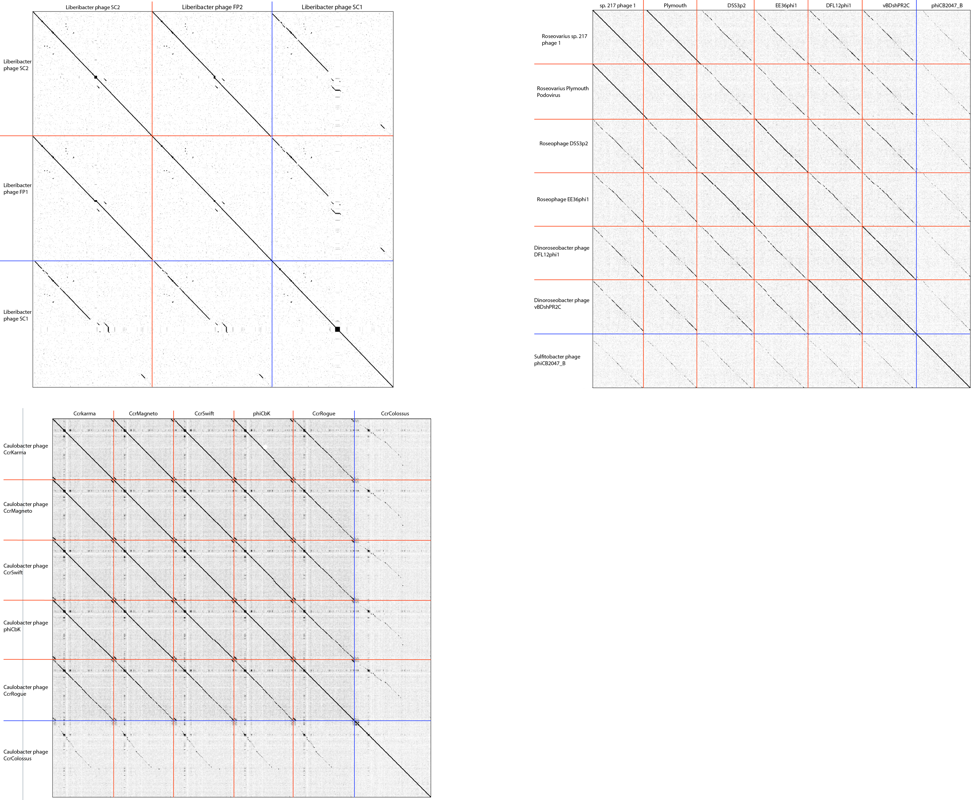

Supplement: Fig. S1 — Nucleotide sequence dot plots from members of three separate clusters showing the presence of subclusters in clusters B (upper-left panel), M1 (upper-right panel), and P (bottom panel). [file spectrum.02827-23-s0002.tiff]

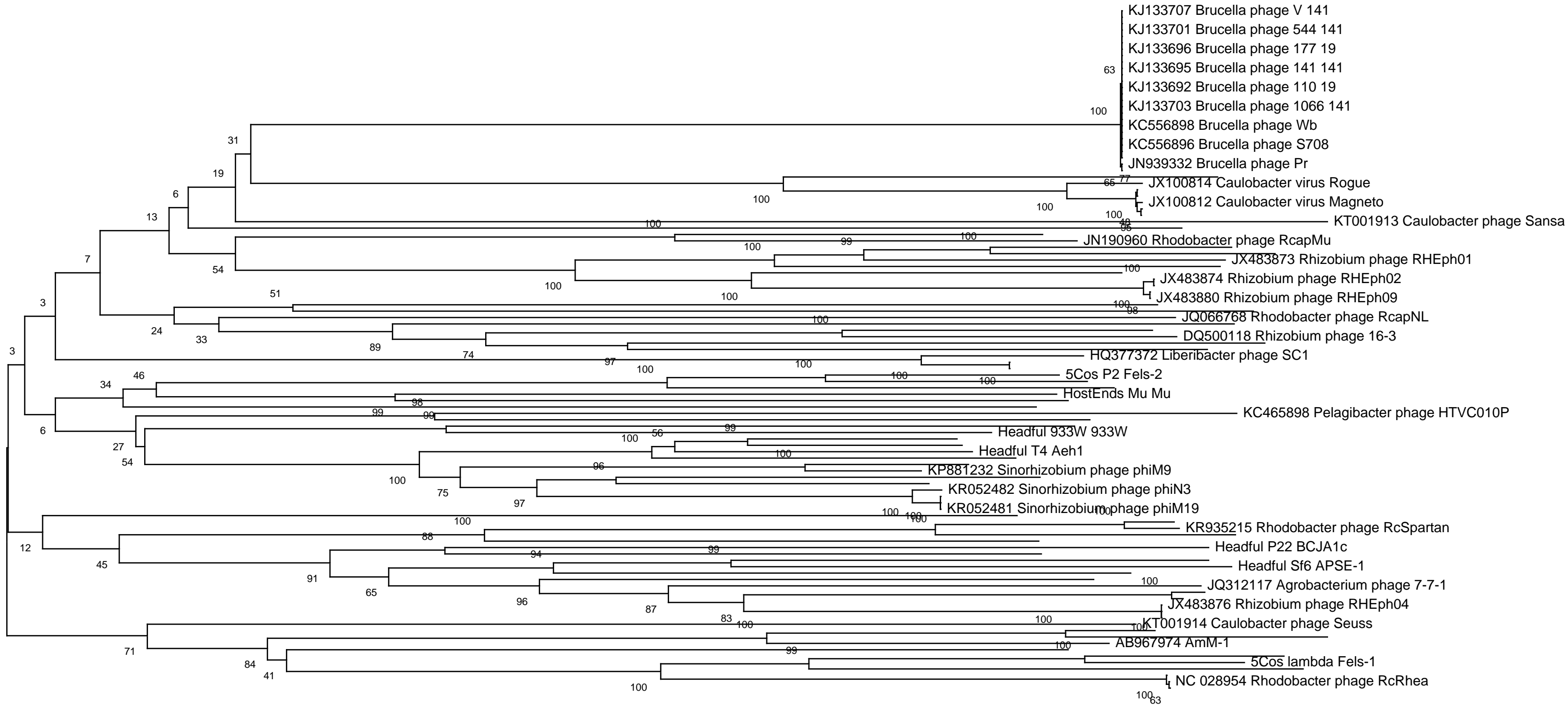

0.20

Supplement: Fig. S2 — Phylogenetic tree of the amino acid sequences from the terminase protein of phages that infect alphaproteobacteria and terminase sequences from phages with well-characterized packaging mechanisms. Scale bar represents % nucleotide diversity. [file spectrum.02827-23-s0003.pdf]
